# Identification of crucial genes based on expression profiles of hepatocellular carcinomas by bioinformatics analysis

**DOI:** 10.7717/peerj.7436

**Published:** 2019-08-08

**Authors:** Ze-Kun Liu, Ren-Yu Zhang, Yu-Le Yong, Zhi-Yun Zhang, Can Li, Zhi-Nan Chen, Huijie Bian

**Affiliations:** Fourth Military Medical University, Department of Cell Biology, National Translational Science Center for Molecular Medicine, Xi’an, Shaanxi, China

**Keywords:** Hepatocellular carcinoma, Gene expression profile, Crucial genes, Methylation, ESR1

## Abstract

Hepatocellular carcinoma (HCC) is one of the most heterogeneous malignant cancers with no effective targets and treatments. However, the molecular pathogenesis of HCC remains largely uncertain. The aims of our study were to find crucial genes involved in HCC through multidimensional methods and revealed potential molecular mechanisms. Here, we reported the gene expression profile GSE121248 findings from 70 HCC and 37 adjacent normal tissues, all of which had chronic hepatitis B virus (HBV) infection, we were seeking to identify the dysregulated pathways, crucial genes and therapeutic targets implicated in HBV-associated HCC. We found 164 differentially expressed genes (DEGs) (92 downregulated genes and 72 upregulated genes). Gene ontology (GO) analysis of DEGs revealed significant functional enrichment of mitotic nuclear division, cell division, and the epoxygenase P450 pathway. Kyoto Encyclopedia of Genes and Genomes (KEGG) analysis showed that the DEGs were mainly enriched in metabolism, cell cycle regulation and the p53 signaling pathway. The Mcode plugin was calculated to construct a module complex of DEGs, and the module was mainly enriched in cell cycle checkpoints, RHO GTPase effectors and cytochrome P450. Considering a weak contribution of each gene, gene set enrichment analysis (GSEA) was performed, revealing results consistent with those described above. Six crucial proteins were selected based on the degree of centrality, including NDC80, ESR1, ZWINT, NCAPG, ENO3 and CENPF. Real-time quantitative PCR analysis validated the six crucial genes had the same expression trend as predicted. Furthermore, the methylation data of The Cancer Genome Atlas (TCGA) with HCC showed that mRNA expression of crucial genes was negatively correlated with methylation levels of their promoter region. The overall survival reflected that high expression of NDC80, CENPF, ZWINT, and NCAPG significantly predicted poor prognosis, whereas ESR1 high expression exhibited a favorable prognosis. The identification of the crucial genes and pathways would contribute to the development of novel molecular targets and biomarker-driven treatments for HCC.

## Introduction

Hepatocellular carcinoma (HCC) is the sixth most frequent malignant cancer and the fourth-leading cause of cancer-related death worldwide (Bray et al., 2018). Patients with hepatitis B virus (HBV), hepatitis C virus (HCV), alcohol consumption and aflatoxin exposure exhibit an increased risk of liver cancer (El-Serag, 2002). Tumorigenesis is largely ascribed to genetic changes, including structure variations, genetic copy number variants, single nucleotide variants and small insertions and deletions (Lengauer, Kinzler & Vogelstein, 1998). Although surgical resection, liver transplantation and sorafenib have prolonged the survival of HCC patients, high postoperative recurrence and drug resistance remain risk factors that confer a worse prognosis (Wang et al., 2015). Traditional treatment strategies for HCC are not optimal. Thus, an increased understanding of the molecular mechanism of HCC initiation and occurrence is of great importance and can be helpful to explore novel therapeutic targets for HCC.

Integration of molecular phenotypes at different levels of HCC subtypes can provide a genetic map for surgically resected tumors and provide information on genomic, transcriptomic,and epigenetic regulation (Hoshida et al., 2009). These different molecular subtypes represent diverse biologic backgrounds of HCC patients and have potential effects on drug treatment options and prognosis. The Gene Expression Omnibus (GEO) and The Cancer Genome Atlas (TCGA) public databases, with a relatively comprehensive gene expression data of HCC, provide the opportunity for the bioinformatics mining (Jiang & Liu, 2015). For example, a comprehensive analysis of the genomic and epigenomic landscape of HCC identified significant genes altered in tumors, such as APOB, MDM4, MET, TERT and so on (Cancer Genome Atlas Research Network, 2017). Lee et al. used GEO and TCGA databases to find COL1A1 was upregulated in HCC and may as a potential therapeutic target of HCC initiation and progression (Ma et al., 2019). CD5L, SLC22A10, UROC1, and SPP2, were identified in alcohol-related HCC datasets (Zhang et al., 2019). To date, numerous dysregulated and somatic mutations of genes and signaling pathways have been identified that play critical roles in HCC initiation and progression (Kan et al., 2013), including TP53 (Kan et al., 2013), UBE3C (Jiang et al., 2014), EGFR (Jang et al., 2017), SHP-1 (Wen et al., 2018) and JAK/STAT (Kan et al., 2013). The study indicated that chromosomal instability might be a driving force of TP53 inactivation and CCND1 and FGF19 amplifications (Wang et al., 2013). In addition, posttranscriptional modification of genes also has a cumulative effect on promoting HCC. The study found that hypomethylation of the CD147 promoter region accelerated HCC progression and was associated with worse prognosis (Kong et al., 2011). Unfortunately, most of the driver genes (CTNNB1, TP53, AXIN1, TERT and ARID2) previously identified do not have clinical applications (Schulze et al., 2015). Despite these findings, tumor heterogeneity and the small samples used in studies on HCC limit our understanding of HCC, and we are currently unable to genotype patients based on individual genome variations for drugs and early diagnosis.

In the study, we downloaded raw data for the gene expression profile GSE121248 from the GEO database, which included 70 HBV-induced HCCs and 37 adjacent normal tissues. A Robust Multichip Averaging (RMA) normalization algorithm was used to screen differentially expressed genes (DEGs). We further utilized the centrality method of degree to identify crucial proteins and build the significant modules of molecular complex clusters in the protein-protein interaction (PPI) network. We also validated mRNA expression of six significantly crucial genes by real-time PCR, revealing results consistent with the public database analysis. Methylation of crucial genes in HCC was also assessed. Survival probability analysis of the six crucial genes was assessed using TCGA data.

## Materials & Methods

### Microarray data

The raw microarray data of GSE121248, including 70 HCC tissues and 37 adjacent normal tissues, were downloaded from the GEO database (http://www.ncbi.nlm.nih.gov/geo/), which was an openly available database. Another two datasets of GSE25097 and GSE22058 were used as the testing sets to verify our results.

### Data processing and identification of DEGs

The bioconductor package (version3.7, http://www.bioconductor.org/), affy package (version1.58.0, http://bioconductor.org/packages/affy/) and the RMA algorithm (Irizarry et al., 2003) were used for data preprocessing and normalization in R (version 3.5.1, R Core Team, 2018). The *t* test methods of the limma package (version 3.38.2, http://bioconductor.org/packages/limma/) (Ritchie et al., 2015) were used to identify DEGs in HCC tissues compared to adjacent normal tissues. Values of log Fold Change >2.0 or log Fold Change <−2.0 and *P*–value <0.05 served as the cut-off criteria.

### Functional annotation of DEGs

Gene Ontology (GO) analysis was applied using the Database for Annotation, Visualization and Integrated Discovery (DAVID, version 6.8, https://david.ncifcrf.gov/) to explore the biological processes, cellular components and molecular function in which the DEGs were involved (Ashburner et al., 2000). GeneAnswers (version 2.24.0, http://bioconductor.org/packages/GeneAnswers/) was a Kyoto Encyclopedia of Genes and Genomes (KEGG)-based R package that not only provided the annotation of biochemistry pathways associated with diseases and drugs but also facilitated visualization of the signaling pathways enriched by DEGs (Feng et al., 2010).

### Gene set enrichment analysis of HCC

Gene Set Enrichment Analysis (GSEA) (version 3.0, the broad institute of MIT and Harvard, http://software.broadinstitute.org/gsea/downloads.jsp) was performed between HCC and adjacent normal tissues to investigate the biological characteristic of HCC (Subramanian et al., 2005). In detail, the ‘collapse data set to gene symbols’ was set to true, the ‘permutation type’ was set to phenotype, the ‘enrichment statistic’ was set to weighted, and the Signal2Noise metric was used for ranking genes. GSEA calculated a gene set Enrichment Score (ES) that analyzed genes were enriched in the biological signal conduction on the MsigDB (Molecular Signatures Database, http://software.broadinstitute.org/gsea/msigdb). And the meandiv normalization method was used for enrichment scores in the gene sets. In this study, 1,000 gene of permutations were set to generate a null distribution for enrichment score in the hallmark gene sets and functional annotation gene sets. The gene sets database used for enrichment analysis were mainly including ‘h.all.v6.2.symbols.gmt’, ‘c2.cp.kegg.v6.2.symbols.gmt’, ‘c2.cp.reactome.v6.2.symbols’ and ‘c5.bp.v6.2.symbols’. Nominal *P*–value <0.05, FDR <0.25 and gene set size >100 were defined as the cut-off criteria.

### PPI network and module analysis

The PPI network was analyzed using the STRING database for *Homo sapiens* (Szklarczyk et al., 2017). STRING was used to calculate PPI networks of DEGs with a combined score >0.4 as the cut-off criteria. Cytoscape (version3.7.0, https://cytoscape.org/) was used to visualize the network (Shannon et al., 2003). Mcode (version1.5.1, Bader Lab, University of Toronto, http://www.baderlab.org/Software/MCODE/) was a Cytoscape plugin for constructing the protein modulecomplex with a degree cut-off = 2, node score cut-off = 0.2, max.depth = 100, and *k*-core = 2. CytoNCA (version 2.1.6, http://apps.cytoscape.org/download/stats/cytonca/) was used to analyze centrality of protein interaction networks that were representative of potentially crucial proteins in the network (Tang et al., 2015). The crucial proteins were identified based on four different centrality parameters (degree centrality (DC), betweenness centrality (BC), eigenvector centrality (EC) and closeness centrality (CC)). The values of centrality parameters were sorted to select the top 10 ranked proteins, and the proteins were input as the crucial candidates. ClueGO (version 2.5.3, http://apps.cytoscape.org/apps/cluego) and CluePedia (version 1.5.3, http://apps.cytoscape.org/apps/cluepedia) were employed to analyze the KEGG and Reactome pathways.

### Transcriptional expression level and survival analysis of crucial genes on HCC

The mRNA expression of crucial genes of HCC and adjacent normal tissues were evaluated based on the shinyGEO database, which included two HCC datasets GSE25097 (containing of 268 HCC and 243 adjacent normal tissues) and GSE22058 (containing of 96 HCC and 96 adjacent normal tissues) (Dumas, Gargano & Dancik, 2016). The Human Protein Atlas (http://www.proteinatlas.org) was used for protein detection by immunohistochemistry. We performed TCGA methylation data of MEXPRESS database (https://mexpress.be/) to explain why oncogene mRNAs were highly expressed in HCC. The patients overall survival was investigated using TCGA_LIHC data and the clinicopathological features was download by TCGA database (https://www.cbioportal.org/). Statistical significance was considered at *P* < 0.05.

### HCC sample collection and revalidation of mRNA expression of crucial genes

Thirty pairs of HCC and paired adjacent tissues were obtained from Xijing Hospital, Fourth Military Medical University of China. Our study was approved by the Ethical Committee and Institutional Review Board of Fourth Military Medical University (Ethical Application Ref: KY20183288-1). The RNA was extracted using RNA extraction kit II (Omega Bio-tek, GA, USA) and cDNA was synthesized using PrimeScript™ RT reagent Kit (TaKaRaBio, Otsu, Japan). The mRNA expression was assessed in 384-well plates using the CFX connect™ Real-Time System (BIO-RAD, Hercules, CA, USA) with the SYBR Premix Ex Taq II (TaKaRaBio, Otsu, Japan) and primers, and qPCR data were analyzed using ^△△^Ct method with β-actin as the reference gene. Primer sequences were listed in Table S1.

### Statistical analysis

Statistical analysis was performed with SPSS 19.0 software (IBM, Armonk, NY, USA). All the data were presented as mean ± SEM. Overall survival time was calculated by the Kaplan–Meier method and analyzed with the log-rank test. The univariate and multivariate analyses were calculated based on the Cox regression model. The statistical analysis was performed as appropriate by Student’s *t* test and *χ*^2^ test. The *P*-value < 0.05 were considered statistically significant.

## Results

### Identification of DEGs

**Figure 1:**
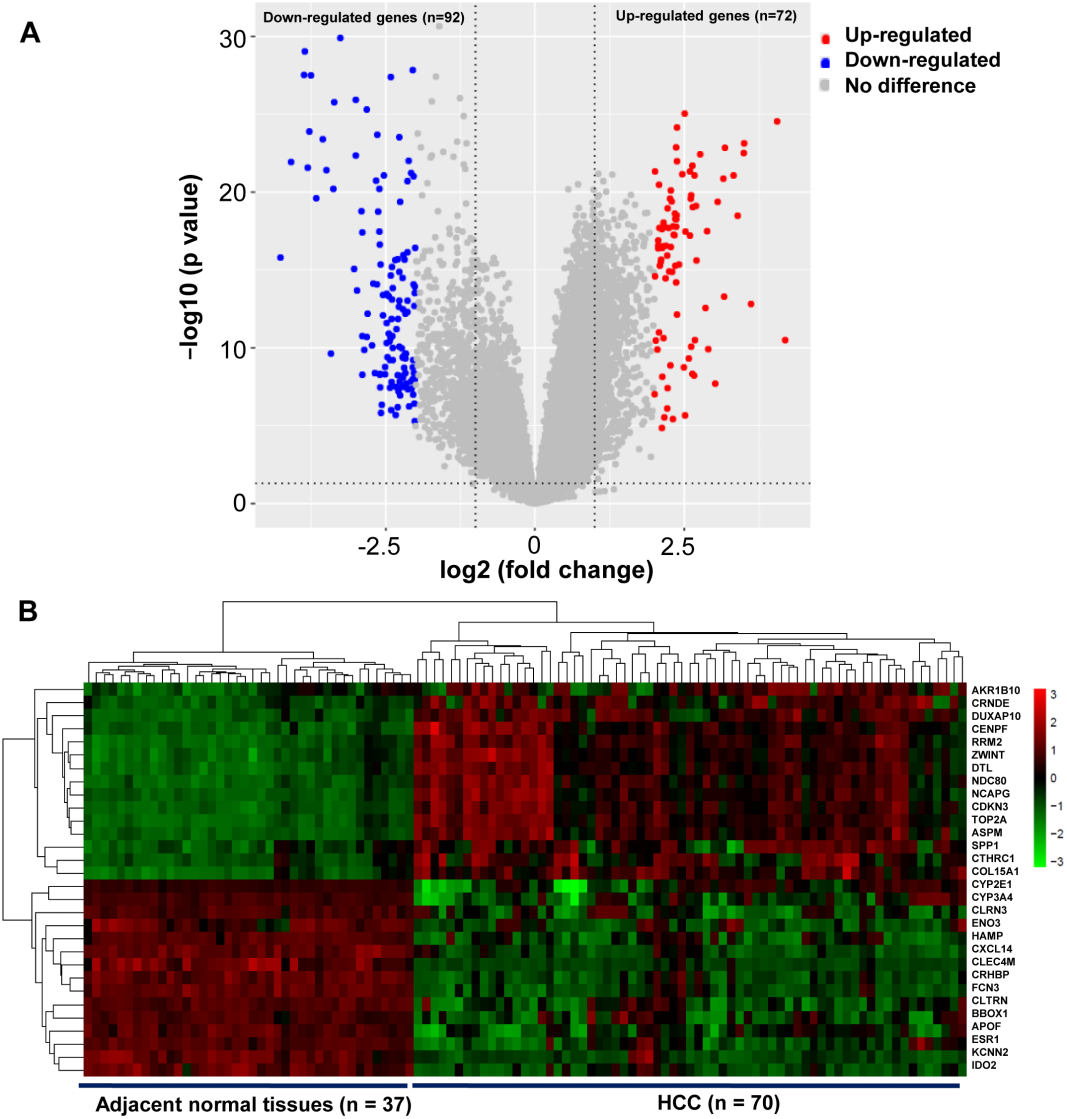
Identification of DEGs in HCC. (A) Volcano plot of gene expression profile. The upper-right red dots represented upregulated genes, and the upper-left blue dots represented downregulated genes. (B) Hierarchical clustering heatmap of the 30 representative DEGs screened on the basis of —fold change— > 2.0 and *P*-value < 0.05. The columns represented the samples, including 37 adjacent normal and 70 HCC tissues. The rows showed that the 30 representative DEGs, including top 15 upregulated expressed genes, and the last 15 downregulated expressed genes in HCC. Red indicated that the expression of genes was relatively upregulated, and the green indicated the expression of genes was relatively downregulated.

A total of 164 DEGs that met the cut-off criteria were identified, including 72 upregulated and 92 downregulated genes (Fig. 1A). The mRNA expression profiles of 30 representative DEGs in 70 HCC and 37 adjacent normal tissues were presented in Fig. 1B.

### Functional annotation analysis of DEGs

**Figure 2:**
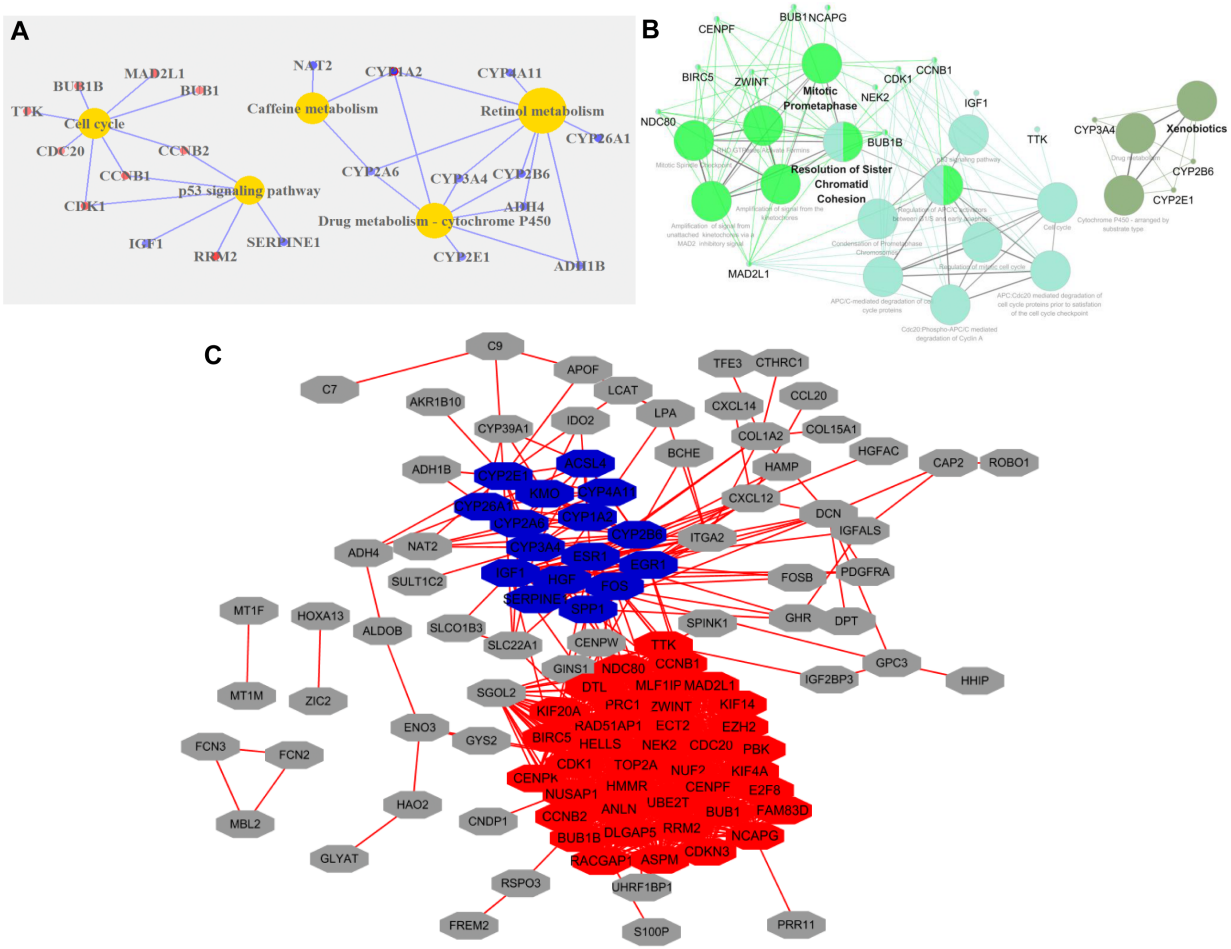
Functional categories of DEGs in HCC. (A) KEGG pathway analysis of DEGs. Red circles represented upregulated genes, and blue represented downregulated genes. (B) KEGG and Reactome pathways analysis of 10 top ranked crucial proteins. The crucial proteins were identified by ClueGO. The colors represented three types of enrichment. (C) The top two modules in the PPI network. The red indicated module 1, and blue indicated module 2.

GO analysis of DEGs revealed that the following GO terms were significantly enriched in condensed chromosome kinetochore (*P* = 1.33E-10), mitotic nuclear division (*P* = 7.45E-10), sister chromatid cohesion (*P* = 1.97E-09), and cell division (*P* = 2.34E-09) (Table S2). KEGG annotation analysis of DEGs in HCC revealed that the pathway mainly including retinol metabolism (*P* = 4.21E-05), drug metabolism-cytochrome P450 (*P* = 5.95E-05), cell cycle (*P* = 2.44E-04), and the p53 signaling pathway (*P* = 5.59E-04) (Fig. 2A).

### Protein–protein interaction network of DEGs

The PPI network of DEGs with 113 nodes (genes) and 933 edges (interactions) was built by STRING. The CytoNCA plugin was used to analyze the centrality of nodes. The top 10 proteins, which were also defined as crucial proteins based on four different centrality parameters, were presented in Table 1, and they included TOP2A, NDC80, ESR1, ZWINT, CENPF, NCAPG, ENO3 and CCNB1. The majority of these crucial proteins participated in mitotic prometaphase (*P* = 1.5E-14) and resolution of sister chromatid cohesion (*P* = 6E-13) (Fig. 2B).

**Table 1:** The top 10 proteins ranked based on the node centrality of the PPI network.

Rank	Degree centrality		Betweenness centrality		Closeness centrality		Eigenvector centrality	
	Gene symbol	Expression in HCC	Gene symbol	Expression in HCC	Gene symbol	Expression in HCC	Gene symbol	Expression in HCC
1	*TOP2A*	up-regulated	*TOP2A*	up-regulated	*TOP2A*	up-regulated	*CCNB1*	up-regulated
2	*NDC80*	up-regulated	*ESR1*	down-regulated	*NDC80*	up-regulated	*CDK1*	up-regulated
3	*CCNB1*	up-regulated	*CYP3A4*	down-regulated	*ESR1*	down-regulated	*NDC80*	up-regulated
4	*CDK1*	up-regulated	*CYP2E1*	down-regulated	*CDKN3*	up-regulated	*ZWINT*	up-regulated
5	*CDKN3*	up-regulated	*NDC80*	up-regulated	*BIRC5*	up-regulated	*BUB1*	up-regulated
6	*ZWINT*	up-regulated	*IGF1*	down-regulated	*CENPF*	up-regulated	*TTK*	up-regulated
7	*TTK*	up-regulated	*CYP2B6*	down-regulated	*CCNB1*	up-regulated	*NCAPG*	up-regulated
8	*BUB1*	up-regulated	*COL1A2*	up-regulated	*CDK1*	up-regulated	*RACGAP1*	up-regulated
9	*CENPF*	up-regulated	*CENPF*	up-regulated	*EZH2*	up-regulated	*BUB1B*	up-regulated
10	*NCAPG*	up-regulated	*ENO3*	down-regulated	*NEK2*	up-regulated	*MAD2L1*	up-regulated

The MCODE plugin was employed to identify the modules in the PPI network. The top 2 significant modules were displayed in Fig. 2C, and the seed of two separate modules are RAD51AP1 and ESR1. KEGG and Reactome pathway analyses revealed that the two modules were mainly related to cell cycle checkpoints (*P* = 7.94E-15), RHO GTPase effectors (*P* = 6.87E-13), and Cytochrome P450 (*P* = 1.37E-12) (Table S3).

### Gene Set Enrichment Analysis of HCC

**Figure 3:**
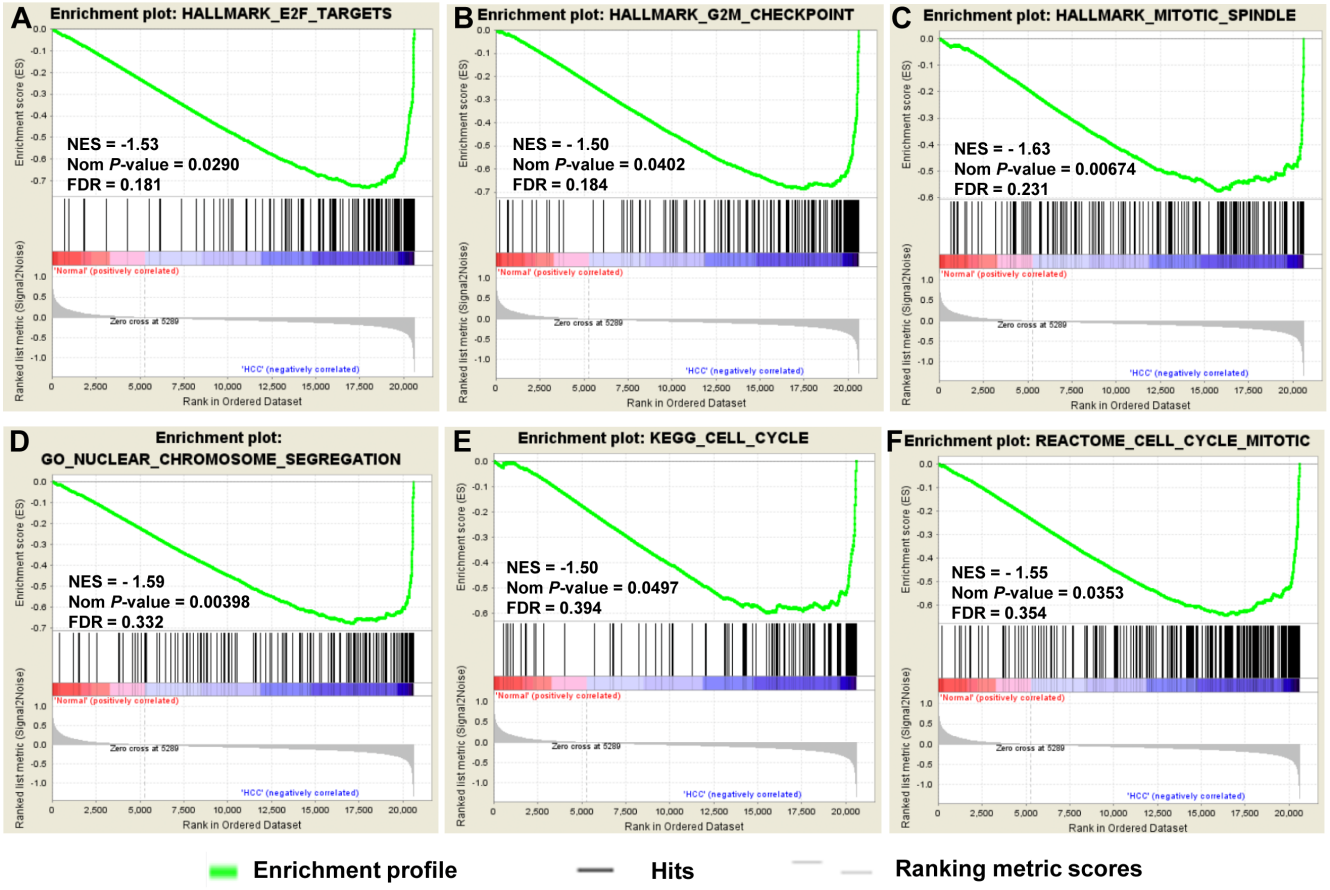
GSEA of gene expression data in HCC. The gene expression profiling identified cell cycle dysregulation of HCC. The enrichment of whole expressed genes were analyzed using GSEA of Hallmark collection database, mainly including ‘E2F_TARGETS’ term (A), ‘G2M_CHECKPOINT’ term (B) and ‘MITOTIC_SPINDLE’ term (C). GO terms (D) and pathway databases of KEGG (E), Reactome (F) analysis demonstrated that the whole expressed genes were enriched in cell cycle progression-related gene sets. The false discovery rates (FDR), the nominal *P*-value and the normalized enrichment score were calculated for each gene set. Each black bar at the bottom of each panel represented a member gene of the respective gene set.

To provide further insight into the whole gene enrichment annotation of HCC and consider the potential role of undifferentiated genes or genes with smaller differences in expression in HCC and adjacent normal tissues, GSEA was performed to explore the gene expression profile based on the GO terms and pathway databases of KEGG, Reactome and Hallmark gene sets. The result revealed that enrichment for three of six gene sets were significant at *FDR* <0.25, including genes involved in cell cycle and chromosome segregation (Fig. 3).

### Validation of mRNA expression of crucial genes

**Figure 4:**
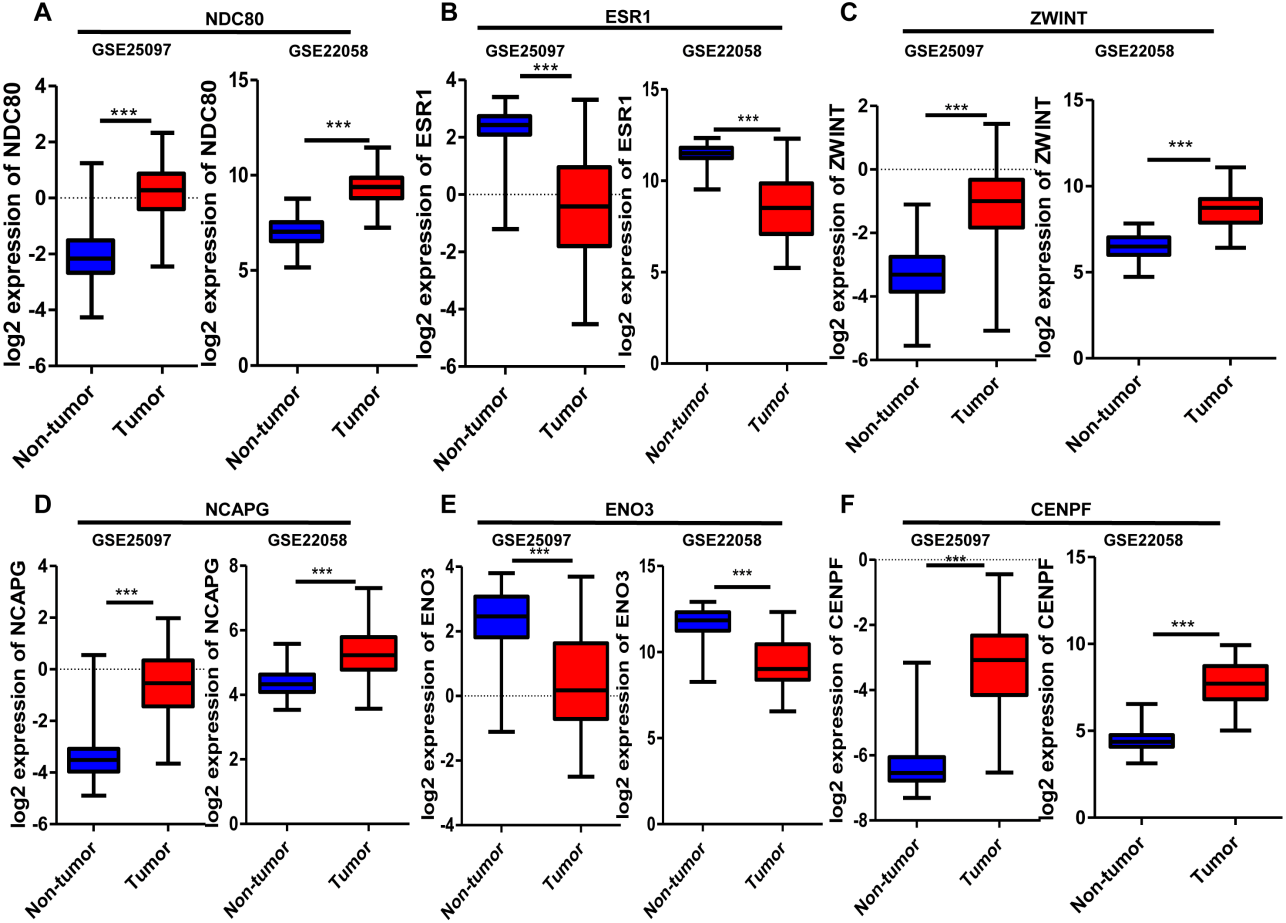
Validation of differential expression of six crucial genes in GEO public datasets. Increased expression of (A) NDC80, (C) ZWINT, (D) NCAPG and (F) CENPF in HCC. Reduced expression of (B) ESR1 and (E) ENO3 in HCC, *** *P* < 0.001.

**Figure 5:**
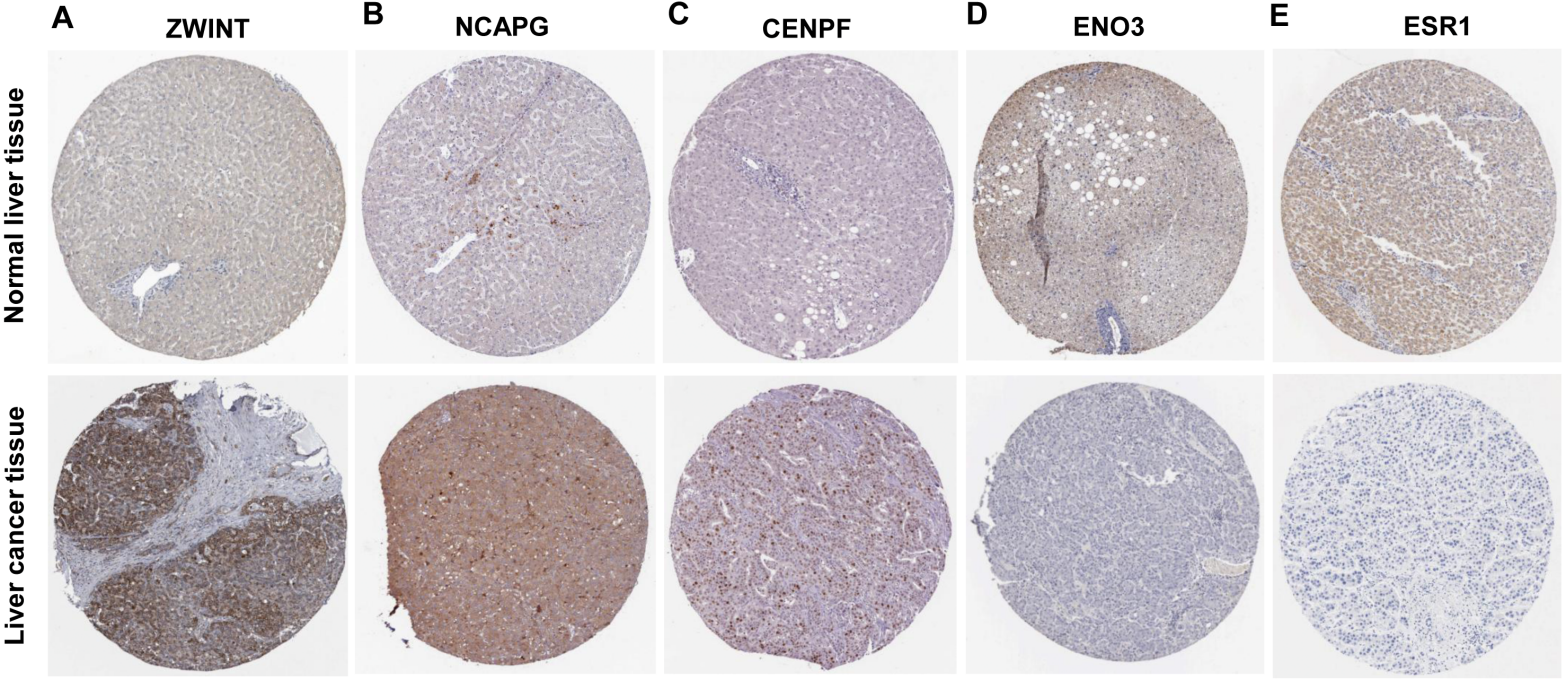
Protein expression of the crucial genes in liver cancer and normal tissues based on The Human Protein Atlas database. (A) ZWINT, (B) NCAPG and (C) CENPF proteins were strongly upregulated in liver cancer tissues compared with normal tissues. (D) ENO3 and (E) ESR1 proteins were downregulated in liver cancer tissues compared with normal tissues.

**Figure 6:**
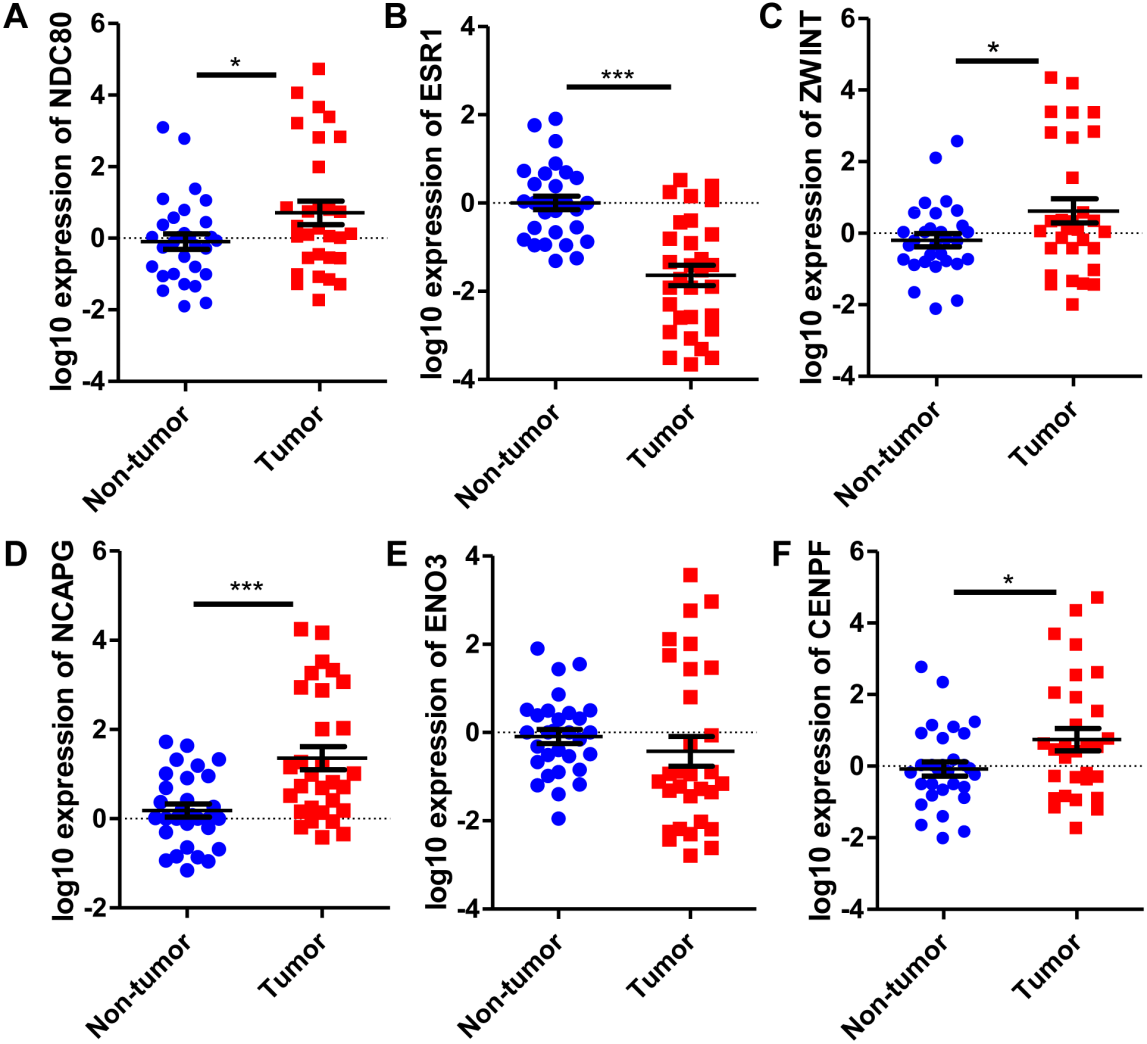
Revalidation of six crucial genes in 30 pairs of HCC and paired non-tumor tissues. Quantitative real-time PCR results for the six crucial genes. (A) NDC80; (B) ESR1; (C) ZWINT; (D) NCAPG; (E) ENO3; (F) CENPF. Expression of these crucial genes was standardized against β-actin expression. The statistical analysis was calculated by the Student’s *t* test. *P < 0.05, ***P < 0.001.

Six significantly crucial genes, NDC80, ESR1, ZWINT, NCAPG, ENO3 and CENPF, were validated in two other HCC datasets (GSE25097 and GSE22058). The results also revealed that NDC80, ZWINT, NCAPG and CENPF were significantly upregulated in HCC, and ESR1 and ENO3 were downregulated in HCC (Fig. 4). Then, immunohistochemistry staining validated from the Human Protein Atlas database showed that ZWINT, NCAPG and CENPF protein expression were strongly upregulated in liver cancer tissues compared with normal tissues (Figs. 5A–5C). ENO3 and ESR1 protein were downregulated in liver cancer tissues compared with normal tissues (Figs. 5D & 5E). The protein expression of NDC80 was absent in the Human Protein Atlas database. Consistent with database analyses, the six crucial gene were successfully validated by qPCR in 30 paired human HCC and adjacent normal tissues, and the results revealed no difference with the analysis results of microarray profiling with the exception of ENO3 (Fig. 6).

The relationship between the methylation and mRNA expression of crucial genes were respectively validated by TCGA data to identify oncogenes with highly expressed mRNA in HCC. The results showed that mRNA expression of crucial genes was negatively correlated with methylation levels. The genes highly expressed or hypomethylated were mostly involved in HCC. Hypermethylated/poorly expressed crucial genes in HCC tended to exhibit high expression in adjacent normal tissues (Table 2).

**Table 2:** Validation of the relationship between methylation and mRNA expression of crucial genes in TCGA.

Crucial gene	mRNA expression in HCC		Methylation status in HCC			
	mRNA expression	*P*-value	Methylation	Position	Pearson correlation	*P*-value
*NDC80*	High expression	<2.2E-16	Hypomethylation	chr18:2571606	−0.150	1.64E-03
				chr18:2571747	−0.282	<2.2E-16
*ZWINT*	High expression	<2.2E-16	Hypomethylation	chr10:58121299	−0.142	0.03
*NCAPG*	High expression	<2.2E-16	Hypomethylation	chr4:17812759	−0.236	2.26E-11
				chr4:17813558	−0.544	<2.2E-16
*CENPF*	High expression	<2.2E-16	Hypomethylation	chr1:214776904	−0.243	1.48E-12
				chr1:214777596	−0.304	1.60E-14
				chr1:214780880	−0.174	6.27E-11
				chr1:214781456	−0.154	1.38E-04
				chr1:214781663	−0.217	1.21E-05
				chr1:214788192	−0.150	7.40E-07
*ESR1*	Low expression	<2.2E-16	Hypermethylation	chr6:152002969	−0.139	2.58E-02
				chr6:152003038	−0.142	5.60E-05
*ENO3*	Low expression	7.46E-14	Hypermethylation	chr17:4850943	−0.160	1.39E-03
				chr17:4851808	−0.146	5.41E-12
				chr17:4852013	−0.139	8.45E-04
				chr17:4852349	−0.118	9.08E-12
				chr17:4854754	−0.705	8.20E-05
				chr17:4855149	−0.604	2.24E-06

### Kaplan–Meier survival analysis of crucial genes in HCC patients

**Figure 7:**
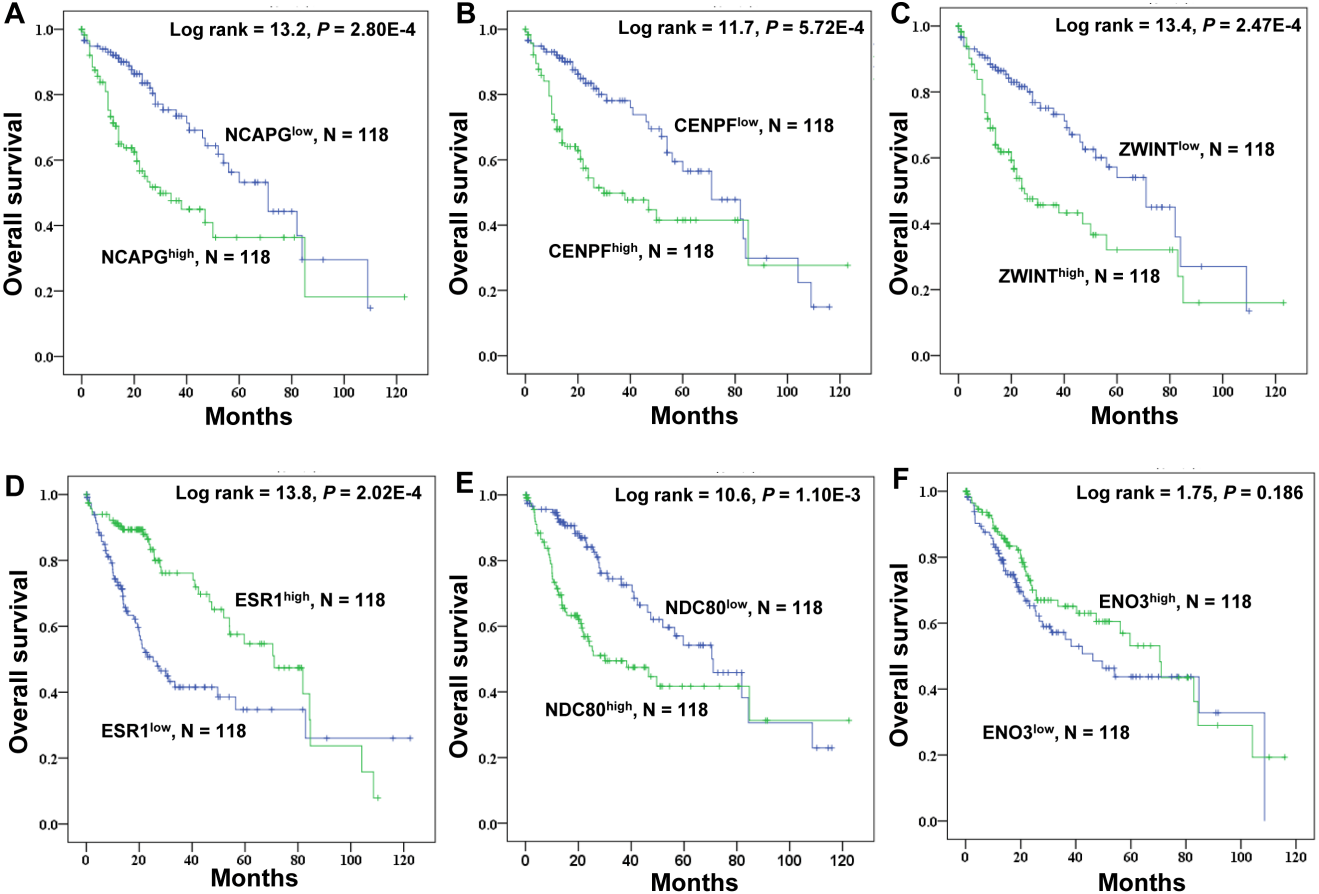
Kaplan–Meier survival analysis of 118 liver cancer samples using the TCGA database. The prognostic survival analysis of the six crucial genes in HCC patients. (A) NCAPG; (B) CENPF; (C) ZWINT; (D) ESR1; (E) NDC80; (F) ENO3. The green lines represent patients with high gene expression, and blue lines represent patients with low gene expression.

TCGA data were used to explore the relationship between the crucial genes and the survival of HCC patients, and high expression of NDC80 (*P* = 1.10E-3), NCAPG (*P* = 2.80E-4), CENPF (*P* = 5.72E-4) and ZWINT (*P* = 2.47E-4) significantly correlated with worse survival probability for HCC patients. ESR1 (*P* = 2.02E-4) exhibited the opposite trend, and ENO3 was not a significant marker of survival prognosis (*P* = 0.186) (Fig. 7). There we focused a crucial tumor suppressor gene, ESR1. The RNA expression data from the TCGA_LIHC cohorts revealed that increased ESR1 mRNA expression correlated significantly with gender, age, serum AFP, TNM stage, tumor recurrence and tumor differentiation (Table S4). Univariate analysis also revealed that the crucial genes ESR1 expression was significantly correlated with overall survival and recurrence (Table S5). Multivariate analysis was performed using all of the variables that were identified as significant by univariate analysis. The result showed that ESR1 mRNA expression was an independent prognostic indicator for overall survival and recurrence, and other features of patients with HCC were also analyzed (Table S5).

### The mRNA expression of ER related receptor and regulatory genes in HCC

**Figure 8:**
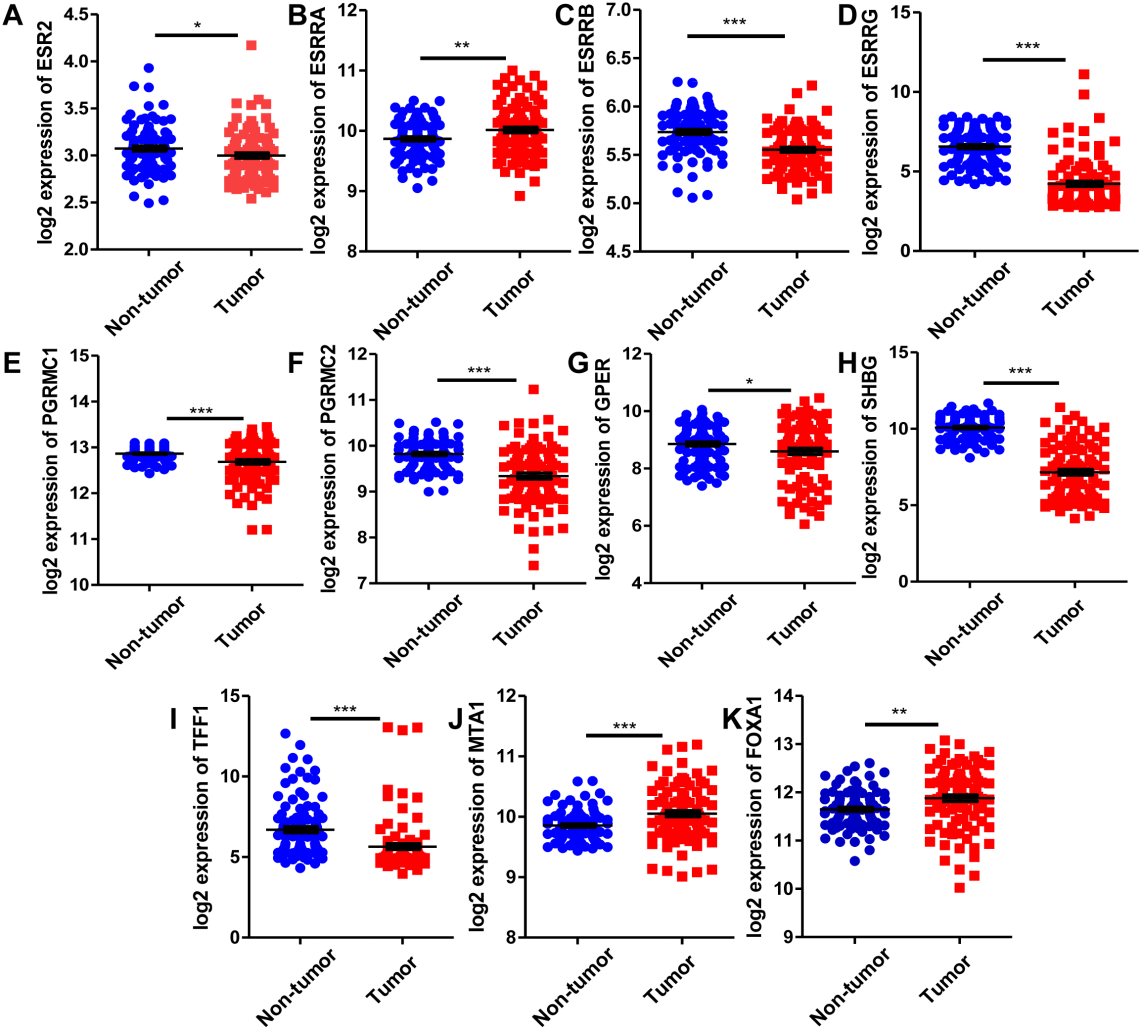
Comparison of mRNA expression of ER-related regulon genes in human HCC tumor tissues and adjacent tissues using GEO database GSE22058 (*n* = 96). The mRNA expression of sex hormone-related genes in HCC tissues relative to the adjacent non-tumor tissues. (A) ESR2; (B) ESRRA; (C) ESRRB; (D) ESRRG; (E) PGRMC1; (F) PGRMC2; (G) GPER; (H) SHBG. The mRNA expression of ER regulatory genes in HCC tissues relative to the adjacent non-tumor tissues. (I) TFF1; (J) MTA1; (K) FOXA1. *P < 0.05, **P < 0.01, ***P < 0.001.

**Figure 9:**
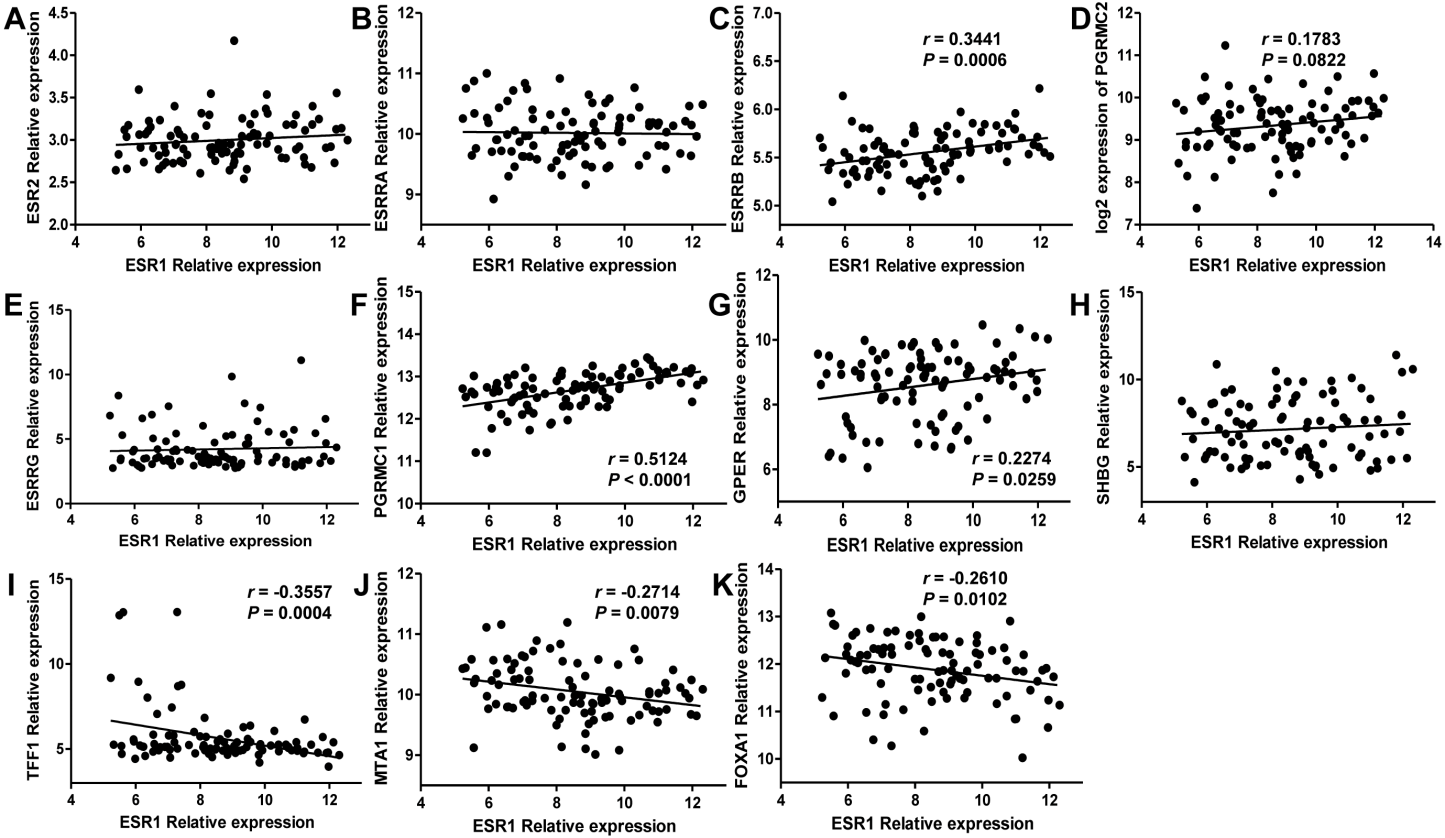
Correlation of ESR1 and ER regulon genes in HCC tissues based on database GSE22058 (*n* = 96). The correlations between ESR1 mRNA expression and sex hormone-related genes expression were analyzed (A–H), and the correlations between ESR1 mRNA expression and ER regulatory genes were analyzed (I–K).

Since estrogen plays a protective role in liver cancer by binding to estrogen receptors, we also further investigated the expression of ER (estrogen receptor) regulon genes in HCC tissues compared with adjacent tissues and the correlation between ER regulon genes and ESR1 using the public GEO database (GSE22058). The mRNA expression levels of sex hormone- related genes (ESR2, ESRRB, ESRRG, PGRMC1, PGRMC2, GPER, SHBG) and ER regulatory gene, TFF1 in HCC tissues were significantly lower than those of adjacent tissues, whereas the expression of estrogen-related receptor gene, ESRRA and ER regulatory genes (MTA1 and FOXA1) were higher in tumor tissues (Fig. 8). In HCC tissues, ESR1 mRNA level was positively correlated with ESRRB (*r* = 0.3441, *P* = 0.0006), PGRMC1 (*r* = 0.5124, *P* < 0.0001) and GPER (*r* = 0.2274, *P* = 0.0259) (Fig. 9). Moreover, ESR1 mRNA level was negatively correlated with ER regulatory genes MTA1 (*r* =  − 0.2714, *P* = 0.0079) and TFF1 (*r* =  − 0.3557, *P* = 0.0004) (Fig. 9).

## Discussion

In this study, we systematically analyzed gene expression profiles of HCC, including 70 HCC and 37 adjacent normal tissues, using bioinformatics methods. A total of 167 DEGs were identified, including 72 upregulated and 92 downregulated genes. The GO terms indicated that DEGs were enriched in mitotic nuclear division, sister chromatid cohesion, cell division, oxidoreductase activity and condensed chromosome kinetochore. KEGG pathway analysis revealed that the DEGs were enriched in the cell cycle, and the p53 signaling pathway and in metabolism. To eliminate individual genetic biases, the enrichment of entire genes was performed through GSEA analysis. Whole genes were mainly enriched in cell cycle and chromosome segregation, which was consistent with the above enrichment results.

One of the distinguishing characteristics in cell division progression is the segregation of sister chromatids. Incorrect segregation of chromosomes could be caused by abnormal regulation or damage of the spindle assembly checkpoint, which subsequently increased chromosome instability and aneuploidy and accelerated tumor progress (Carter et al., 2006). RB deficiency induced chromosomal instability to promote liver tumorigenesis (Mayhew et al., 2007). In addition, the cytochrome P450 family of proteins were downregulated in HCC based on microarray analysis. Jiang et al. confirmed that CYP3A5 was a suppressor by regulating mTORC2/AKT to antagonize the malignant phenotype of HCC (Jiang et al., 2015). RYO et al. found that CYP3A4 downregulation was related to a poor prognosis for 92 HCC patients (Ashida et al., 2017). Therefore, abnormal regulation of the cell cycle together with chromosome segregation or inactivation of some cytochrome P450 proteins could contribute to hepatogenesis.

In recent years, bioinformatics mining has been widely used for identifying tumor features and novel diagnosis markers (Jiang & Liu, 2015). For example, Agarwal et al. found that 59 kinases related genes were overexpressedin HCC, and furthermore predicted overall four distinct HCC subtypes and each subtype represented unique gene expression profiling, pathway enrichment and prognosis using TCGA dataset (Agarwal et al., 2017). Wang et al. analyzed the potential biomarks for HCC by integrated GEO and TCGA datasets and identified AKR1B10 as a novel biomarker (Wang et al., 2018). Yin et al. showed that the genes involved in the G2/M checkpoint may act as biomarkers at the early HCC (Yin, Chang & Xu, 2017). In addition, another study found that hub genes TOP2A, PCNA and AURKA with low mutation frequency involved in HCC by bioinformatics analysis (Xing, Yan & Zhou, 2018). However, their study only analyzed a profile, and only identified the gene with a high node degree using the module analysis. In addition, their selected genes were validated in terms of genetic alteration method or the Kaplan–Meier plotter database. Our study combined the results of MCODE, CytoNCA and methylation modification for the identification of crucial genes. Moreover, we validated the results by the other three databases GSE25097, GSE22058, TCGA_LIHC and RT-PCR, and used clinical features and prognosis of the patients to evaluate the importance of crucial gene, thus promoting the reliability of our results.

According to degree centrality and molecular module analysis of differentially expressed proteins in the PPI network, the crucial proteins including TOP2A, NDC80, ESR1, CCNB1, ZWINT, CENPF, ENO3 and NCAPG were identified. Subsequently, the molecular module of DEGs was constructed to understand the closely related subgroups, and the seed of two separate modules was displayed as RAD51AP1 and ESR1. Furthermore, NDC80, ZWINT, NCAPG, and CENPF were upregulated in HCC as demonstrated by qPCR and associated with poor prognosis. ESR1 was a favorable prognostic factor. ZWINT, NCAPG and CENPF were frequently identified in the related enrichment sets by GSEA analysis.

Interestingly, we found that ESR1 mRNA expression was downregulated in HCC, and ESR1 mRNA expression was negatively correlated with methylation levels of its promoter region. ESR1 was associated with a favorable prognostic factor, and may as a potential tumor suppressor in HCC. Epidemiological studies showed that the prevalence rate of HCC was lower in female than in male patients (El-Serag & Rudolph, 2007). Chronic hepatic disease progressed more slowly to cirrhosis and HCC in female than in male patients (Shimizu, 2003). Therefore, the estrogens may play a protective role in the progression of HCC. To our knowledge, ESR1 (estrogen receptor *α*), which encodes the estrogen receptor, has a role in hormone binding and activation of transcription. Hishida et al. previously reported that the expression level of ESR1 was downregulated in 24 (50%) patients with HCC (Hishida et al., 2013). Estrogen inhibited the transcription of HBV by upregulating ER *α*, which changed the interaction with HNF4 *α* to alter binding to HBV enhancer I (Wang et al., 2012). Studies have reported that estrogen exerted protective effects against HCC through inhibiting IL-6 production (Naugler et al., 2007). Moreover, we identified ESR1 mRNA level was negatively correlated with estrogen-related regulatory genes TFF1 and MTA1 using HCC dataset GSE22058.

In addition, NDC80 is a kinetochore complex componentthat can organize and stabilize microtubule-kinetochore interactions and is required for proper chromosome segregation. NDC80 was overexpressed in many solid cancers, and silencing of NDC80 inhibited cell cycle and cell proliferation (Meng et al., 2015). The small molecule SM15 bound to microtubules and NDC/Hec1 to control tumor growth through microtubule stabilization (Ferrara et al., 2018). Ying et al. confirmed that ZWINT interacted with cell cycle-related proteins to promote HCC proliferation (Ying et al., 2018). Liu et al. (2018) demonstrated that NCAPG played an oncogenic role in the development of HCC. Shahid et al. (2018) found that CENPF expression was increased in higher risk prostate cancer patients. ENO3 encoded beta-enolase, which was involved in glycolysis and gluconeogenesis, and little information had been reported about the function of ENO3 (Kong et al., 2016). Our study utilized multidimensional bioinformatics methods to seek and validate network crucial genes in association with HCC from the database of TCGA and GEO. We identified six crucial genes (NDC80, ESR1, ZWINT, NCAPG, ENO3 and CENPF) based on the degree of centrality and module analysis of DEGs network and studied the significance of the expression pattern of the crucial genes for overall survival of patients with HCC. Moreover, we used qRT-PCR to validate our bioinformatics results: NDC80, ZWINT, NCAPG and CENPF mRNA expressions were significantly upregulated in HCC, and ESR1 and ENO3 were downregulated in HCC. Furthermore, by TCGA data, mRNA expression of crucial genes were negatively correlated with methylation levels of their promoter region. Additionally, ESR1 expression correlated significantly with clinicopathological variables, indicating that it may be a crucial tumor suppressor gene of HCC. The crucial genes we identified may be useful biomarkers for diagnosis and prognosis of HCC, even if further experimental validations are needed to confirm the findings.

##  Supplemental Information

10.7717/peerj.7436/supp-1 Figure S1 The box plot for the GSE121248 data before normalization (above, ‘Original’), and the box plot for the data after normalization (below, ‘RMA’)

10.7717/peerj.7436/supp-2 Table S1 Primers for real-time PCR

10.7717/peerj.7436/supp-3 Table S2 GO terms of differentially expressed genes in HCC

10.7717/peerj.7436/supp-4 Table S3 Pathways enriched by the top two modules

10.7717/peerj.7436/supp-5 Table S4 Clinical features and ESR1 mRNA expression in HCC patients

10.7717/peerj.7436/supp-6 Table S5 Univariate and multivariate analysis of factors associated with overall survival and recurrence in the mRNA expression study of ESR1

10.7717/peerj.7436/supp-7 Table S7 Identification of DEGs

10.7717/peerj.7436/supp-8 Table S7 GO and KEGG results

10.7717/peerj.7436/supp-9 Table S8 Two modules of PPI network

10.7717/peerj.7436/supp-10 Table S9 Centrality of nodes of PPI network

10.7717/peerj.7436/supp-11 Table S10 Clinicopathological characteristics of TCGA_LIHC

10.7717/peerj.7436/supp-12 Table S11 Raw data of PCR results of six crucial genes

## Additional Information and Declarations

### Microarray Data Deposition

The following information was supplied regarding the deposition of microarray data: Data is available at the Gene Expression Omnibus database, accession numbers: GSE25097, GSE22058 and GSE121248.

### Data Availability

The following information was supplied regarding data availability: The raw measurements are available in the Supplemental Tables.
