# Supplementary material for: Identification of crucial genes based on expression profiles of hepatocellular carcinomas by bioinformatics analysis
Source: PeerJ. 2019 Aug 8;7:e7436. doi: 10.7717/peerj.7436 (PMC6689388; doi:10.7717/peerj.7436)
Supplement: Table S1 [file peerj-07-7436-s002.docx]

Supplementary Table 1. Primers for real-time PCR.

| Gene | Forward primer | Reverse primer |
| --- | --- | --- |
| *NDC80* | TCAAGGACCCGAGACCACTTA | GGGAGCTTGTAGAGATTTCATGG |
| *ESR1* | CCCACTCAACAGCGTGTCTC | CGTCGATTATCTGAATTTGGCCT |
| *ZWINT* | AGGACACTGCTAAGGGTCTCG | GCCTCTACGTGCTCCCTGTA |
| *ENO3* | TATCGCAATGGGAAGTACGATCT | AAGCTCTTATACAGCTCTCCGA |
| *CENPF* | CTCTCCCGTCAACAGCGTTC | GTTGTGCATATTCTTGGCTTGC |
| *NCAPG* | GAGGCTGCTGTCGATTAAGGA | AACTGTCTTATCATCCATCGTGC |
| *β-actin* | AGCGAGCATCCCCCAAAGTT | GGGCACGAAGGCTCATCATT |
