# Supplementary material for: Identification of crucial genes based on expression profiles of hepatocellular carcinomas by bioinformatics analysis
Source: PeerJ. 2019 Aug 8;7:e7436. doi: 10.7717/peerj.7436 (PMC6689388; doi:10.7717/peerj.7436)
Supplement: Table S2 [file peerj-07-7436-s003.docx]

Supplementary table 2. GO terms of differentially expressed genes in HCC

| Category | Term | Count | % | *P*-Value | FDR | Fold enrichment |
| --- | --- | --- | --- | --- | --- | --- |
| GOTERM_BP | GO:0007067~mitotic nuclear division | 17 | 10.69 | 7.45E-10 | 1.19E-06 | 7.62 |
|  | GO:0007062~sister chromatid cohesion | 12 | 7.55 | 1.97E-09 | 3.16E-06 | 12.96 |
|  | GO:0051301~cell division | 19 | 11.95 | 2.34E-09 | 3.75E-06 | 6.04 |
|  | GO:0019373~epoxygenase P450 pathway | 5 | 3.14 | 1.70E-05 | 0.0272 | 30.89 |
|  | GO:0007094~mitotic spindle assembly checkpoint | 5 | 3.14 | 2.65E-05 | 0.0424 | 27.80 |
| GOTERM_CC | GO:0000777~condensed chromosome kinetochore | 12 | 7.55 | 1.33E-10 | 1.66E-07 | 16.65 |
|  | GO:0030496~midbody | 11 | 6.92 | 1.12E-07 | 1.40E-04 | 10.29 |
|  | GO:0000776~kinetochore | 9 | 5.66 | 3.38E-07 | 4.21E-04 | 13.41 |
|  | GO:0005576~extracellular region | 33 | 20.75 | 2.15E-06 | 0.00268 | 2.47 |
|  | GO:0000775~chromosome, centromeric region | 7 | 4.40 | 7.22E-06 | 0.00900 | 14.82 |
| GOTERM_MF | GO:0016705~oxidoreductase activity | 7 | 4.40 | 9.16E-06 | 0.0128 | 14.20 |
|  | GO:0020037~heme binding | 9 | 5.66 | 2.40E-05 | 0.0331 | 7.60 |
|  | GO:0019825~oxygen binding | 6 | 3.77 | 5.01E-05 | 0.0692 | 14.76 |
|  | GO:0005506~iron ion binding | 9 | 5.66 | 5.29E-05 | 0.0732 | 6.80 |
|  | GO:0004497~monooxygenase activity | 6 | 3.77 | 1.39E-04 | 0.192 | 11.96 |
