# Supplementary material for: Identification of crucial genes based on expression profiles of hepatocellular carcinomas by bioinformatics analysis
Source: PeerJ. 2019 Aug 8;7:e7436. doi: 10.7717/peerj.7436 (PMC6689388; doi:10.7717/peerj.7436)
Supplement: Table S3 [file peerj-07-7436-s004.docx]

Supplementary table 3. Pathways enriched by the top two modules

| Module | Term | *P-*value | Genes |
| --- | --- | --- | --- |
| 1 | Cell Cycle Checkpoints | 7.94E-15 | *BIRC5, BUB1, BUB1B, CCNB1, CCNB2, CDC20, CDK1, CENPF, CENPK, CENPU, MAD2L1, NDC80, NUF2, ZWINT* |
|  | RHO GTPase Effectors | 6.87E-13 | *BIRC5, BUB1, BUB1B, CDC20, CENPF, CENPK, CENPU, KIF14, MAD2L1, NDC80, NUF2, PRC1, ZWINT* |
|  | p53 signaling pathway | 4.04E-05 | *CCNB1, CCNB2, CDK1, RRM2* |
|  | Kinesins | 5.33E-04 | *KIF20A, KIF4A, RACGAP1* |
| 2 | Cytochrome P450-arranged by substrate type | 1.37E-12 | *CYP1A2, CYP26A1, CYP2A6, CYP2B6, CYP2E1, CYP3A4, CYP4A11* |
|  | Retinol metabolism | 2.05E-10 | *CYP1A2, CYP26A1, CYP2A6, CYP2B6, CYP3A4, CYP4A11* |
