# Supplementary material for: Identification of crucial genes based on expression profiles of hepatocellular carcinomas by bioinformatics analysis
Source: PeerJ. 2019 Aug 8;7:e7436. doi: 10.7717/peerj.7436 (PMC6689388; doi:10.7717/peerj.7436)
Supplement: Table S4 [file peerj-07-7436-s005.docx]

Supplementary table 4. Clinical features and ESR1 mRNA expression in HCC patients

| Clinicopathological Variables | *ESR1* mRNA level | | *P* value |
| --- | --- | --- | --- |
|  | Low (n=177) | High (n=180) |  |
| Gender |  |  |  |
| Male | 108 | 134 | 0.007 |
| Female | 69 | 46 |  |
| Age |  |  |  |
| ≥ 50 years | 129 | 163 | *P*<0.001 |
| < 50 years | 48 | 17 |  |
| Serum AFP |  |  |  |
| ≤ 20(ng/ml) | 47 | 97 | *P*<0.001 |
| > 20(ng/ml) | 83 | 46 |  |
| N/A | 47 | 37 |  |
| TNM stage# |  |  |  |
| Ⅰ | 73 | 103 | 0.003 |
| Ⅱ-Ⅳ | 104 | 77 |  |
| Tumor recurrence |  |  |  |
| Yes | 89 | 79 | 0.037 |
| No | 57 | 82 |  |
| N/A | 31 | 19 |  |
| Tumor differentiation |  |  |  |
| G1-G2 | 93 | 135 | *P*<0.001 |
| G3-G4 | 84 | 45 |  |

AFP, α-fetoprotein; TNM stage, Tumor & Node & Metastasis stage.

#Clinical typing of tumors was performed using the tumor node metastasis (TNM) classification system of the American Joint Committee on Cancer (AJCC) and the Union for International Cancer Control (UICC) (7th ed.)
