# Supplementary material for: Identification of crucial genes based on expression profiles of hepatocellular carcinomas by bioinformatics analysis
Source: PeerJ. 2019 Aug 8;7:e7436. doi: 10.7717/peerj.7436 (PMC6689388; doi:10.7717/peerj.7436)
Supplement: Table S5 [file peerj-07-7436-s006.docx]

Supplementary table 5. Univariate and multivariate analysis of factors associated with overall survival and recurrence in the mRNA expression study of ESR1

| **Clinicopathological characteristics** | **Univariate analysis** | | | | | | **Multivariate analysis** | | | | | |
| --- | --- | --- | --- | --- | --- | --- | --- | --- | --- | --- | --- | --- |
|  | **Overall survival** | | | **Recurrence** | | | **Overall survival** | | | **Recurrence** | | |
|  | **Exp(B)** | **95% CI** | ***P* value** | **Exp(B)** | **95% CI** | ***P* value** | **Exp(B)** | **95% CI** | ***P* value** | **Exp(B)** | **95% CI** | ***P* value** |
| **Gender (Female vs Male)** | **1.198** | **0.835-1.721** | **0.327** | **1.118** | **0.809-1.546** | **0.499** |  |  |  |  |  |  |
| **Age (<50 vs ≥50 years)** | **1.233** | **0.776-1.957** | **0.375** | **0.986** | **0.668-1.455** | **0.986** |  |  |  |  |  |  |
| **AFP (≤20 vs >20ng/ml)** | **0.748** | **0.588-0.950** | **0.017** | **0.872** | **0.698-1.091** | **0.231** | **0.730** | **0.577-0.922** | **0.008** | **NA** | | |
| **TNM stage (ⅠvsⅡ-Ⅳ)** | **2.566** | **1.800-3.657** | ***P*<0.001** | **2.287** | **1.658-3.153** | ***P*<0.001** | **2.337** | **1.629-3.353** | ***P*<0.001** | **2.264** | **1.640-3.127** | ***P*<0.001** |
| **Histologic grade (G1-2 vs G3-4)** | **1.117** | **0.779-1.602** | **0.546** | **1.100** | **0.805-1.503** | **0.549** |  |  |  |  |  |  |
| ***ESR1* expression (Low vs High)** | **0.539** | **0.377-0.771** | **0.001** | **0.648** | **0.478-0.877** | **0.005** | **0.526** | **0.365-0.758** | **0.001** | **0.659** | **0.486-0.894** | **0.007** |

Note: Univariate analysis and Multivariate analysis, Cox proportional hazards regression model; AFP, α-fetoprotein; 95% CI, 95% confidence interval.
